# Events as Elements of Physical Observation: Experimental Evidence

**DOI:** 10.3390/e26030255

**Published:** 2024-03-13

**Authors:** J. Gerhard Müller

**Affiliations:** Department of Applied Sciences and Mechatronics, Munich University of Applied Sciences, D-80335 Munich, Germany; gerhard.mueller@hm.edu

**Keywords:** physical measurement, information gain, event generation, physical action, energy dissipation, space–time expansion

## Abstract

It is argued that all physical knowledge ultimately stems from observation and that the simplest possible observation is that an event has happened at a certain space–time location $\vec{X}=\left(\vec{x},t\right)$. Considering historic experiments, which have been groundbreaking in the evolution of our modern ideas of matter on the atomic, nuclear, and elementary particle scales, it is shown that such experiments produce as outputs streams of macroscopically observable events which accumulate in the course of time into spatio-temporal patterns of events whose forms allow decisions to be taken concerning conceivable alternatives of explanation. Working towards elucidating the physical and informational characteristics of those elementary observations, we show that these represent hugely amplified images of the initiating micro-events and that the resulting macro-images have a cognitive value of 1 bit and a physical value of $W_{obs}=E_{obs}\tau_{obs}\gg h$. In this latter equation, $E_{obs}$ stands for the energy spent in turning the initiating micro-events into macroscopically observable events, $\tau_{obs}$ for the lifetimes during which the generated events remain macroscopically observable, and h for Planck’s constant. The relative value $G_{obs}=W_{obs}/h$ finally represents a measure of amplification that was gained in the observation process.

## 1. Introduction

In this paper, we are concerned with the problem of gaining information about nature by performing physical experiments. In order to introduce this subject, we sketch in Section 2 three historic experiments which were ground-breaking in the evolution of theories which form the background of our current understanding of matter on the atomic, nuclear, and elementary particle scales. These are the Rutherford scattering experiments of Geiger and Marsden [1], which proved the nuclear nature of atoms [2,3]; the double-slit experiments performed with photons and all kinds of corpuscular matter, which proved the dual nature of matter [4,5,6,7]; and the cloud, bubble, and streaming chamber experiments [8,9,10] in high-energy physics, which led to the discovery of the standard model of elementary particles [11]. In the past, these experiments were conceived and carried out with the aim of producing macroscopically observable phenomena which allow conceivable alternatives of explanation to be distinguished that had been discussed at their times of invention. 

Regarding these key experiments as questions posed to nature, it is interesting to note that all questions are answered in the form of transient effects which are localized in space and time, and which accumulate over time into spatio-temporal patterns of events which allow decisions to be taken concerning conceivable alternatives of explanation. Turning to those elementary observations, it is clear that the events of observation need to involve a great deal of amplification to turn them into macroscopic images of those initiating events between matter and experimental equipment that had occurred on the microscale. A second relevant observation is that the events of observation are meaningless in the sense that they do not yield any information other than that that an event has happened or not at a certain space–time location $\vec{X}=(\vec{x},t)$. As such elementary observations yield binary decisions between two alternatives, the experimental answers produced by these key experiments resemble messages sent over digital communication channels in which complex and meaningful messages are made up from individual, but otherwise meaningless, bits [12,13,14]. 

While the traditional interpretations of the above key experiments tacitly assumed that particles, waves, and fields are primary entities of physical reality, and that the events of observation are secondary effects produced by the interactions of those primary entities with the experimental equipment, this historic mindset was more recently challenged by the idea that all physical entities at their core are information-theoretic in origin. This latter idea, which was raised by John Archibald Wheeler [15] and aphoristically termed “it from bit”, has raised a vivid controversy between the traditional “bit from it” and the more recent “it from bit” approaches [16,17]. 

In view of this controversy, it appeared to be relevant to re-consider the three key experiments with an informational perspective in mind. In the present paper, we concentrate on those elementary observations that, in the course of time, build up the experimental answers produced by the three key experiments. After a brief review of these experiments in Section 2, we discuss in Section 3 and Section 4 the informational and physical characteristics of those elementary observations that show up as macroscopically observable events. On the whole, this discussion reveals that the elements of physical observation have a double nature in that these are abstract pieces of information on the one hand, and concrete physical entities on the other hand. As physical entities, elementary observations reveal as pieces of physical action, produced at the expense of generating entropy. With this conclusion in mind, elementary observations appear as another manifestation of Landauer’s original conclusion [18,19,20,21], namely that “information is physical” at its origin. The processes of generating and erasing elementary observations and of assigning meaning to discrete patterns of observable events will be discussed in forthcoming papers [22,23]. 

## 2. The Three Key Experiments

**Figure 1:**
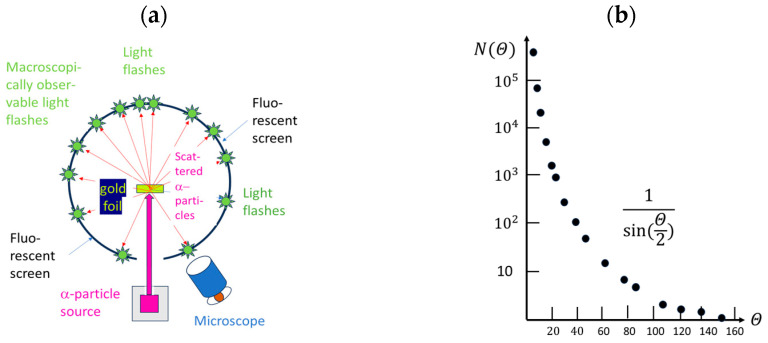
(**a**) Sketch of a Rutherford scattering experiment [1] which proved the nuclear constitution of atomic matter [3]. Alpha-particle scattering from a gold foil produces flashes of light on the fluorescent screen (green stars), whose angular distribution can be interpreted as evidence that most of the mass of Au atoms is concentrated in small volumes with linear dimensions on the order of 10^−12^ cm [3]. (**b**) Angular distribution of light flashes as observed in the original work of Geiger and Marsden in 1913 [1].

**Figure 2:**
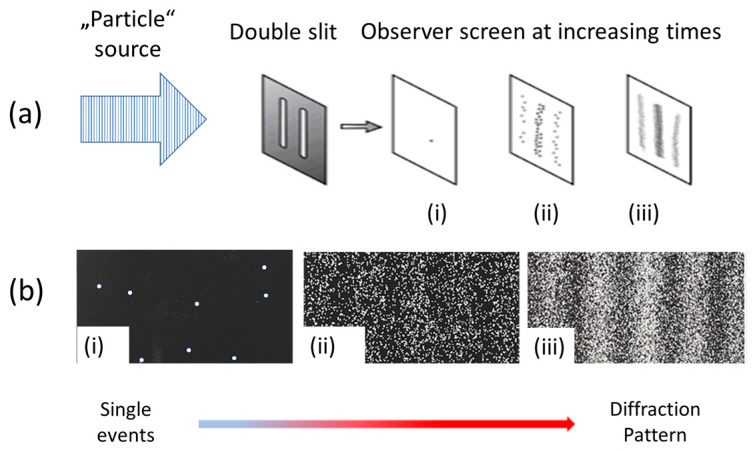
(**a**) Matter in the form of photons, electrons, atoms, and molecules is passed through the double-slit arrangements in (**a**) in one-by-one manner [4,5,6,7].; (**b**) After having passed through the double-slit arrangement in (**a**), the transmitted “particles” interact with a photographic screen on the right, producing macroscopically observable events which accumulate in the form of diffraction patterns after more and more “particles” have been processed through the experimental arrangement in (**a**). Screen shots at increasingly larger times are shown in subfigures (i); (ii); (iii) [28].

**Figure 3:**
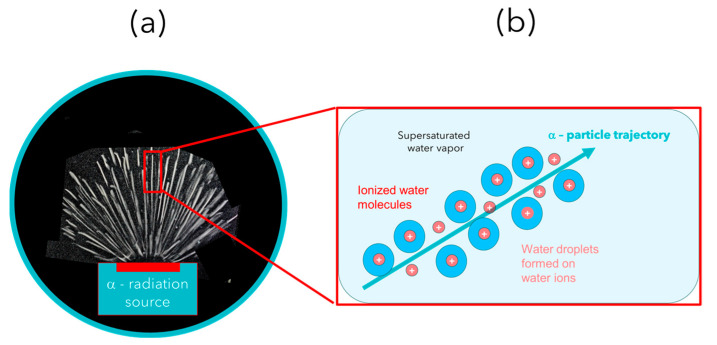
(**a**) α-particle trajectories emerging from an α-particle source immersed inside a cloud chamber [8,29]; (**b**) schematic view of a cloud chamber track of water droplets condensed on water ions formed along the α-particle trajectories [29].

After the above preliminary considerations, we turn to a more in-depth discussion of those experiments which have been accepted as ground-breaking in the evolution of physical sciences. For the sake of discussion, these historic experiments are sketched in Figure 1, Figure 2 and Figure 3 below.

Moving from top to bottom, these examples show the Rutherford scattering experiments that convincingly demonstrated the nuclear nature of atoms [1,2,3] and rejected the earlier “plum pudding model” of atoms proposed by J. J. Thompson [24]. In this way, the road towards the Bohr theory of the hydrogen atom [25] and the modern quantum theories of Heisenberg [26] and Schrödinger [27] were paved. 

The double-slit experiments [4,5,6,7], on the other hand, confirmed the assumption of a wave–particle duality underlying the Heisenberg [26] and Schrödinger [27] pictures of the atom. 

The cloud- [8], bubble- [9] and spark-chamber [10] experiments performed in the realm of high-energy physics finally contributed to the discovery of a vast variety of elementary particles, which led to the standard model of elementary particles [11].

## 3. Differences and Commonalities between the Three Key Experiments

Considering the above experiments, these share in common that all of them address processes that occur at length and time scales much too small to be directly observable. The key motivation of all these experiments, consequently, was producing macroscopically observable images of those unobservable micro-phenomena. 

Depending on the kind of physical questions asked, the experimental arrangements take very different forms. Whereas the Rutherford experiment was intended to measure the momentum transfers to α-particles that occur deep inside the electrostatic fields that surround atomic nuclei, the double-slit experiments addressed interference phenomena and the issue of wave–particle duality while the streaming chamber experiments were designed to reveal particle trajectories with the aim of deriving kinetic energies and momenta of nuclear reaction products. 

Concurrent with the architectural differences between the key experiments, the spatio-temporal patterns of events take very different forms. These differences, however, disappear when matter is made to interact with the respective experimental arrangements in a one-by-one manner and when the emerging experimental outputs are monitored as they emerge in the course of time. Looked at as functions of time, all experiments produce phenomena that are localized in space and time, and which are macroscopically observable, i.e., either directly visible by unaided eyes—or at least through some kind of optical instrument such as a microscope, as was used in the Geiger–Marsden experiments [1].

**Figure 4:**
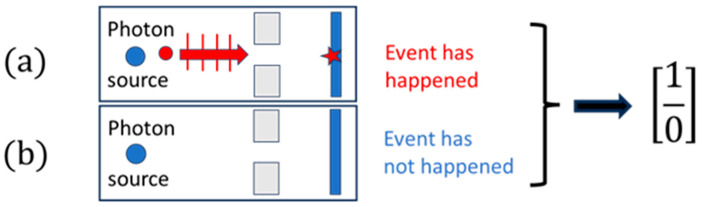
(**a**) A single photon moving from source to fluorescent screen through a narrow slit, either in the form of a particle or in an undulatory manner as a wave; (**b**) no passage of a photon during the observational time period. Elementary observations of this kind produce an information gain equivalent to one binary digit or bit.

As none of these individual observations form neither an angular distribution of scattering events, nor a diffraction pattern, nor a particle trajectory, the observation of each of these individual events does not yield any other information other than that that an event has happened at a certain space–time location or not. As observation or lack of observation of a single event within an observational time interval decides a simple yes/no alternative, each of these single events has a cognitive value of exactly one bit. This idea of making an elementary observation and of choosing between binary alternatives is pictorially represented in Figure 4. There, a photon is sketched that is moving from the source towards a fluorescent screen through a narrow gap. As, on its way from the source to the fluorescent screen, no observation can be made that would allow us to decide whether the photon is moving along a straight-line particle trajectory or in an undulatory manner as a wave, the observation of a single light flash on a fluorescent screen does not allow any other conclusion to be drawn other than that that an event has happened. 

Collecting many of such elementary observations, complex multi-bit messages are produced. In a Rutherford scattering experiment, for instance, angular distributions of sufficiently large numbers of scattered particles can be acquired that allow a decision to be made between scattering in nuclear electric force fields with 1r, 1r2, or hard sphere potentials [2,3,23]. Similarly, distinctions can be made between wave phenomena occurring at different wavelengths and with different arrangements of slits and screens [4], or between particles moving with different momenta through a given magnetic field [8,9,10].

## 4. Emergence and Erasure of Elementary Observations 

**Figure 5:**
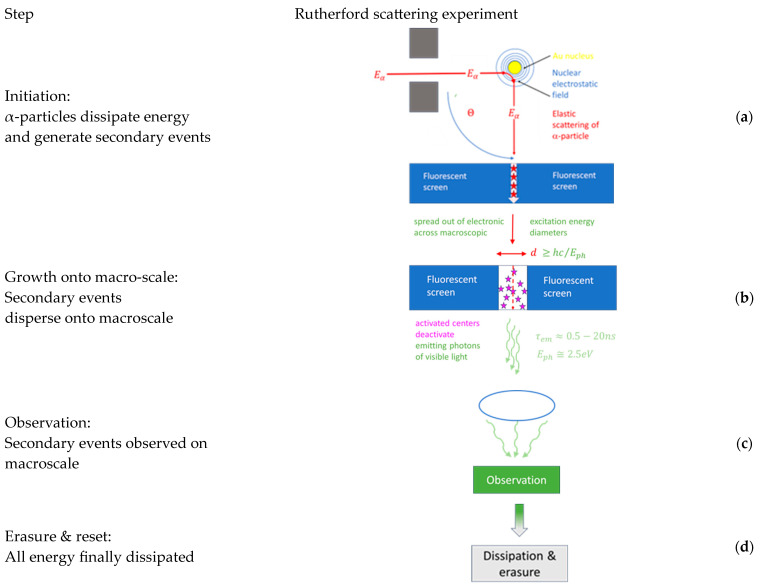
Time-resolved sketch of Rutherford scattering process; sequential steps of information gathering and reset.

**Figure 6:**
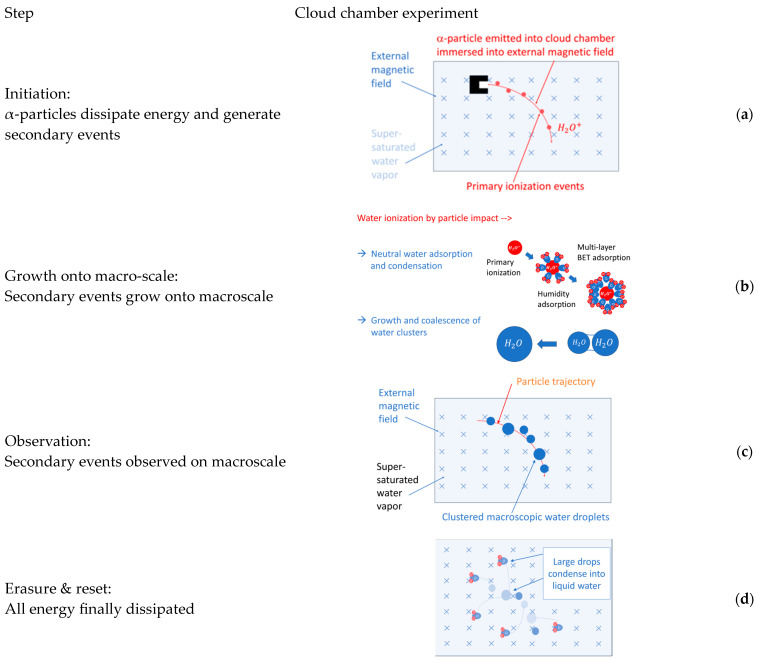
Time-resolved sketch of elementary particle detection in cloud chamber; sequential steps of information gathering and reset.

In the section above, we identified elementary observations as macroscopically observable, binary pieces of information. What has not yet been discussed is how these elementary pieces of information come into existence, and why these occur as temporal transients. In order to move forward into this direction, we re-consider in more detail the processes of Rutherford scattering and of visualizing nuclear particle trajectories in cloud chambers. The time-resolved sketches of these processes in Figure 5 and Figure 6 show that both processes move through a sequence of four steps, namely: initiation, growth, observation, and erasure and reset. All observable effects (light flashes, particle trajectories) that transiently appear on the macro-scale ultimately disappear, as all energy that had produced these effects has finally been dissipated. Such dissipation clearly explains the transient nature of events.

For the sake of clarity, we now move through these four steps, considering Rutherford scattering and the visualization of particle tracks in a cloud chamber sequentially.

### 4.1. Rutherford Scattering 

The initial step in Rutherford scattering is the approach of an α-particle close to the Au nucleus (Figure 5a). With an α-particle energy of $E_{a}≅5\;MeV$, α-particles can approach Au nuclei up to a minimum distance of $r_{min}≅5\times 10^{-12\;}cm,$ which is still larger than the nuclear radius of $R_{Au}≅6\times 10^{-13}\;cm$. The scattering process, therefore, clearly takes place within the strong electrostatic field that surrounds each Au nucleus. During the residence time of $\tau_{int}\approx 2{\;r}_{min}/v_{\alpha }$, where $v_{\alpha }$ is an α-particle velocity of around 5% the speed of light, the physical action associated with the scattering process can be estimated to be $\Delta W≅E_{a}\tau_{min}≅7\;h.$ Changes in physical action on the order of a few Planck units are typical of quantum-mechanical interactions. 

The second part of the initiation process is the absorption of the scattered α-particle inside the ZnS fluorescent layer, as also shown in Figure 5a, and the generation of secondary ionization events. Estimates based on the Bethe–Boch formula [30] show that roughly 80−90 eV of the α-particle’s kinetic energy are transferred into ionization and electronic excitation energy within each mean-free path inside the ZnS layer. With the initial α-particle energy of $E_{a}$ ≅5 MeV and its initial speed of $v_{a}≅$ 0.05c, each scattered α-particle is found to slow down over a length of approximately 20 μm inside the ZnS layer and within a time span of a few picoseconds. During this short time, the scattered α-particles generate roughly $N_{int}=6\times 10^{4}$ secondary ionization events, which form a narrow, straight line of highly electronically excited ZnS material. Due to the large lateral gradients in electronic excitation energy, intense lateral flows of electrons are initiated away from this line. Assuming that, in the ensuing diffusion- and equilibration processes, one single activated center is formed per primary ionization event, $N_{int}$ green-light luminescence photons will ultimately be emitted from the small cylindrical volume in which the α-particle energy had been dissipated (Figure 5b). With the bulk electron mobility in ZnS on the order of $\mu_{n}≅100\;{cm}^{2}/Vs$ [31], lateral diffusion lengths on the order of several micrometers can be estimated. Although the surface diameters of light-emitting ZnS materials of this size are small, these nevertheless amount to multiples of the wavelength of the green luminescence light of $\lambda_{ph}≅0.5\;\mu m$, which allows these light spots to be observed with the help of a microscope (Figure 5c) as actually used in the Geiger–Marsden experiments [1]. 

With this situation in mind, the amount of physical action $W_{obs}$, associated with such green-light-emitting cylindrical volumes (Figure 5c), can be estimated. Assuming that each ionization event ultimately leads to the emission of a green-light photon with an energy of $E_{ph}≅2.5\;eV$ [1] and a luminescence lifetime of $\tau_{lum}≅\;10^{-8}\;s$ [32], a piece of physical action of $W_{obs}≅$ $N_{int}{\;E}_{ph}\;\tau_{lum}$ is generated which amounts to a quantity of 3.5 $\times \;10^{11}$ units of the Planck constant. With a physical action of only 7 units of Planck constant h generated in the initiating scattering process, a huge amount of amplification on the order of $G_{obs}≅5\times 10^{10}$ is inferred to have occurred in the Geiger–Marsden experiment [1]. With this number in place, the macroscopic observability of the initiating microscopic scattering events can be explained. As, finally, after observation, all luminescence light is converted into low-temperature heat (Figure 5d), all of the α-particles’ initial kinetic energy has ultimately been dissipated in the detection process. 

Taking an overall look at the Rutherford scattering experiment, it becomes apparent that each individual scattering event had ultimately become observable by dissipating the kinetic energy of the incoming α-particles. Dissipation in this context means that the huge initial energy of each α-particle was broken down into increasingly smaller packages of energy which were simultaneously spread out over increasingly larger spatial domains. Whereas, in the final stages of dissipation, the temperature of the entire ZnS fluorescence screen was raised by an immeasurably small amount, macroscopic observability of scattering events relies on the fact that, in the process of dissipation, a large number of visible-light photons are intermittently generated as energy dispersion proceeds. As the emitted photons still carry energies much larger than the mean thermal energy of the ZnS lattice atoms, their informational value stands out from the random thermal noise inside the ZnS layer, which ensures their observability [33]. Again, as the energy of these visible light photons is further dissipated in the detection process [34], all kinetic energy of the initiating α-particles is finally dissipated into low-temperature heat, which completely erases all informational value that had originally been carried by the incoming α-particles in the form of kinetic energy [32].

### 4.2. Visualization of Nuclear Particle Tracks 

In the cloud chamber experiment shown in Figure 6a, the initiating micro-event is the emission of an α-particle from the source and the ensuing travel of the particle through an atmosphere of supersaturated water vapor inside the cloud chamber. Again, with the high kinetic energy of each emitted α-particle of around 5 MeV, a large number of secondary ionization events is triggered along each particle’s trajectory. Due to the much lower stopping power of α-particles in super-saturated water vapor [30], however, long tracks of ionization events with lengths on the order of several centimeters are formed [8,29].

After this has happened, the initial ionization is distributed over a large number of $H_{2}O$ molecules, which, because of the auto-protolysis of water [35], results in a large number of $H_{3}O^{+}$ and ${OH}^{-}$ ions. The high electrical fields around each ionized water molecule subsequently encourage neighboring $H_{2}O\;$dipoles to adsorb on the generated water ions, thereby partially shielding the electrostatic field around each molecular ion. After several layers of such dipoles had been adsorbed, the electrical shielding of the $H_{3}O^{+}$ and ${OH}^{-}$ ions has been completed, and, apparently, neutral water droplets had been formed (Figure 6b). With diameters in the range of nanometers, these droplets are still far too small to be visually observable. Once this size range had been reached, a second growth process takes over that grows tiny water droplets into visually observable sizes, and which thus enables the α-particle trajectories to become visually observable. This second stage of droplet growth, also shown in Figure 6b, involves the phenomenon of Ostwald ripening [36]. Ostwald ripening involves the fusion of tiny water droplets into aggregates and the growth of the larger fusion partners at the expense of the smaller ones. In this second phase of growth, the driving force towards larger volumes is the minimization of surface area, and, thus, the reduction in weakly bound surface water molecules at the expense of more tightly bound water molecules inside the bulk. In this way, water droplets with higher condensation energy $Q_{H2O}\left(r\right)\;$are formed with increasing r:

(1) $$Q_{H2O}\left(r\right)=\left(\frac{4\pi }{3}r^{3}\right)\varepsilon_{b}\left[1-3\frac{\gamma_{s}}{\varepsilon_{b}}\frac{1}{r}\right]$$

In this equation, $\varepsilon_{b}=2.26\times 10^{9}\;J/m^{3}$ is the cohesion energy of water [37] and $\gamma_{s}=0.073\;J/m^{2}$ is the surface energy of water [38]. The existence of weakly bound water molecules in the near-surface regions and the desire to reduce their numbers exerts a mechanical pressure on the bulk which leads to enhanced vapor pressure in very small droplets. Very small droplets, therefore, easily and rapidly evaporate, thus re-generating individual H_2_O molecules which are free to adsorb on larger droplets with lower internal pressures. Quantitatively, this excess pressure inside small drops is given by the Kelvin equation [39,40]:

(2) $$p\left(r,T\right)=p_{sat}(T)exp\left[\frac{{2\gamma }_{S}V_{m}}{RT\;r}\right]$$

in which $p_{sat}(T)$ is the vapor pressure over a flat surface at the overall temperature T, R is the universal gas constant, and $V_{m}$ is the molar volume of water.

**Figure 7:**
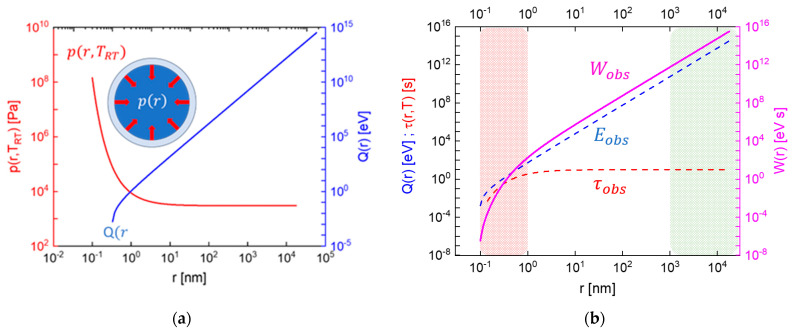
(**a**) Cohesion energy (blue) and internal pressure of water droplets (red) as a function of drop radius. The development of an inside-oriented pressure resulting from the desire to minimize the numbers of weakly bound H_2_O molecules on surfaces is shown in the inset. (**b**) Cohesion energy (blue), evaporative lifetime (red), and observational value (magenta) as a function of drop radius. The colored areas denote the phases of initial growth (red) and of long-lived and macroscopically observable drops that delineate α-particle trajectories.

In Figure 7a, the condensation energy of water droplets $Q_{H2O}\left(r\right)$ is drawn as a function of the drop radius r together with the internal pressure p(r,T) inside these drops. In Figure 7b, the internal pressure data is redrawn, this time, however, with the vapor pressures p(r,T) being converted into time scales τ(r,T) for the evaporation of drops:

(3) $$\tau_{obs}\left(r,T\right)=\tau_{evap}(T,\;r=\infty )exp\left[-\frac{{2\gamma }_{S}V_{m}}{RT\;r}\right]$$

In this mathematical conversion, the assumption has been made that droplets with visually observable sizes evaporate at a time scale of seconds. This latter effect is directly observable in cloud chamber experiments, in which visually observable particle tracks fade away within seconds [8,29]. 

With the condensation energy $Q_{H2O}\left(r\right)$ of the droplets and their evaporative lifetimes $\tau \left(r,T\right)$ in place, the physical action $W_{obs}$ of visible droplets can once again be calculated:

(4) $$W_{obs}\left(r,T\right)=Q_{obs}\left(r\right)\;\tau_{obs}(r,T)\gg h.$$

In Figure 7b, $W_{obs}(r,T_{RT}=300\;K)$ is plotted as a function of the drop radius. As, in the formation of macroscopically visible and relatively long-lived droplets [8,29], a huge number of water molecules is collected, the magnitudes of $W_{obs}(r,T_{RT})$ are much larger than in the Rutherford scattering case. Once measured in units of the Planck constant of $h=4.183\times 10^{-15}\;eVs$, the excessively large values of $W_{obs}(r,T_{RT})$ in the cloud chamber case reflect the fact that these tiny water droplets are observable with un-aided eyes as compared to the tiny light flashes in the Rutherford scattering events, which required additional amplification with the help of an optical microscope [1]. 

Summarizing the considerations about cloud chamber images, some similarity to the case of α-particle scattering can be detected. This similarity is reflected in the formation of primary ionization events in the supersaturated water vapor as highly energetic α-particles are being slowed down, thereby producing drop-initiating $H_{3}O^{+}$ and ${OH}^{-}$ ions. Up to the point of drop-initiating water ions, only the kinetic energies of the incoming α-particles had been dissipated. With the onset of adsorption processes on the initiating ions, energetic resources in the experimental equipment become increasingly involved. Once relatively visible droplets start to cluster into rain drops, supersaturated water vapor is finally converted into the more stable phase of condensed water layers, thus completing the overall dissipation of energy. Again, as in the case of Rutherford scattering of α-particles, the phase of macroscopic visibility occurs in a state of partial equilibration and incomplete but ongoing entropy production. 

### 4.3. Producing Permanent Images of Photon Impacts 

So far, we have avoided the discussion of photographic images of photon impacts on the photographic screens used in the double-slit experiments. Not considering the complexity of the underlying photo-chemical processes, it is immediately clear that much smaller energies in the range of single electron volts are involved in the photo-chemical processes as compared to the huge α-particle energies in the foregoing examples. Instead of the short lifetimes of the intermittently produced visible-light photons in the Geiger–Marsden experiment or the short evaporative lifetimes in the cloud chamber experiments, the photographic detection of photons in the double-slit experiments produces permanent images of the photon impacts. On the level of observational pieces of physical action, $W_{obs}=E_{obs}\tau_{obs}$, the lower energies $E_{obs}$ in photography are largely over-compensated by the huge lifetimes $\tau_{obs}$ of the photographic images. 

## 5. Summary, Conclusions, and Outlook

In this paper, we have considered historical experiments which were groundbreaking in the development of our modern ideas on processes taking place on the length- and timescales of atoms, nuclei, and elementary particles. Regarding these key experiments as questions posed to nature, it has been revealed that these questions are answered in the form of streams of elementary observations which take the form of temporal transients, which are sharply localized in space but still extended enough to be visually observable. Such observable events were identified as binary pieces of information but also endowed with a firm physical existence as pieces of physical action. Considering in some depth the processes of α-particle scattering on atomic nuclei and the visualization of particle trajectories in cloud chambers, the idea has evolved that these elementary observations are pieces of physical action, produced at the expense of energetic resources either carried with the material objects to be detected or contained in the detection equipment itself. With the elementary observations featuring both as abstract pieces of information and as firm pieces of physical reality, the elementary observations produced by the three key experiments appear as another manifestation of Landauer’s initial ideas on memory and switching devices and his conclusive statement of “information is physical” [19]. 

Introducing the quantity $W_{obs}=E_{obs}\tau_{obs}$, where $E_{obs}$ is the energy expended in turning a quantum-mechanical interaction into a macroscopically observable event and $\tau_{obs}$ the lifetime in which an event remains macroscopically observable, a preliminary measure of macroscopic observability has been obtained. Considering the experimental evidence from which this concept was derived, it is revealed that experimentalists have found multiple ways of turning quantum-mechanical interactions on the micro-scale into visually observable events on the macro-scale. Although this is a fascinating proof of experimental creativity, the complexity of the instrumentation and their functional principles are obstacles with regard to accepting elementary observations as theoretically valid concepts. Conceptual devices with simple architectures and easily overseeable physics, such as, for instance, the cylinder–piston-type devices of Szilard engines [41], would allow progress into this direction [22]. 

Another open question concerning large patterns of observable events is the process of assigning meaning to such patterns, i.e., the process of distinguishing between conceivable alternatives of physical explanation. With each elementary observation contributing one single bit, a multi-element patterns would simply constitute a piece of information consisting of N such bits without revealing anything other than a quantitative aspect of the acquired information. Using the acquired information for deciding between alternatives of physical explanation represents an important qualitative aspect of information. Acquiring quality of information requires matching discrete patterns of events onto mental constructs which mathematically feature in the form of continuous functions. In the past, this task has been performed by experimentalists through least-square fitting of experimental data. A formal connection between statistical data matching and quality of information, however, is unknown to the present author and likely outside the realm of the presently accepted measures of Shannon [13] and thermodynamic measures of information [42]. 

## Data Availability

Data is contained within the article.
